# Clinical efficacy of percutaneous closure of patent foramen ovale in children diagnosed with migraine

**DOI:** 10.3389/fcvm.2025.1611338

**Published:** 2025-06-03

**Authors:** Yakun Wang, Xingmiao Liu, Ji Cheng, Dong Li

**Affiliations:** ^1^ Pediatric Cardiovascular Center, Tianjin Children’s Hospital/Tianjin University Children’s Hospital, Tianjin, China; ^2^ Department of Pediatric Neurology, Tianjin Children’s Hospital/Tianjin University Children’s Hospital, Tianjin, China

**Keywords:** percutaneous PFO closure, hidden PFO, migraine, children, retrospective analysis, complete relief of headache

## Abstract

**Objective:**

This retrospective analysis aimed to assess the clinical efficacy of patent foramen ovale (PFO) closure for the treatment of migraine in children.

**Methods:**

Data from 35 children diagnosed with migraine and PFO (pre-intervention transthoracic echocardiography did not detect PFO in some, but saline contrast echocardiography was positive, indicating hidden PFO), admitted to Tianjin Children's Hospital for PFO closure between March 2020 and February 2024, were retrospectively collected and analysed. The efficacy of post-intervention pain relief was evaluated using an 11-point numerical rating scale (NRS-11), headache impact test-6 (HIT-6), and Paediatric Migraine Disability Assessment Score (PedMIDAS), migraine frequency, and duration of each migraine attack.

**Results:**

At the 1-month follow-up after PFO closure, 20 patients (57.1%) achieved complete relief, and 29 (82.9%) had a reduction in migraine attack frequency by >50%. At the 12-month follow-up, 28 patients (80%) achieved complete relief, and 32 (91.4%) had a reduction in migraine attack frequency by >50%. There were no statistically significant differences between the aura and the non-aura groups. All patients exhibited statistically significant improvement (*P* < 0.05) in NRS-11, HIT-6, and PedMIDAS, migraine frequency, and duration of each migraine attack. The children in the aura group had a more significant decrease in NRS-11 than those in the non-aura group at 12-month after the operation (*P* < 0.05). Additionally, there were no statistically significant differences between the two groups in HIT-6, PedMIDAS, and duration of migraine attacks.

**Conclusion:**

Percutaneous PFO closure demonstrated significant clinical efficacy and safety in the treatment of migraine in children.

## 1 Introduction

Migraine is the most common recurrent headache syndrome among children, with approximately 30% of those affected becoming disabled as a result (1). Patent foramen ovale (PFO) is a common congenital disease in children. In most healthy children with isolated PFO, intervention is not required (2). The prevalence of PFO in children with migraines is comparable to that in the general population, with no statistically significant difference observed (35–46.9% vs. 25–25.8%). However, the prevalence of PFO is significantly higher in patients experiencing aura (50%–71.4%) compared to the general population, while it remains similar in those without aura (27%–27.8%) (3, 4).

A large proportion of patients with migraine require long-term medication, and traditional oral migraine therapies, when used chronically, may lead to adverse effects. These include beta-blockers (e.g., propranolol, which may cause fatigue or hypotension), antiepileptic drugs (e.g., topiramate, which may result in cognitive impairment or weight changes), and calcium channel blockers (e.g., flunarizine, which may induce weight gain or drowsiness). Although PFO closure offers a potential new option for migraine prevention—especially in patients with refractory symptoms and a demonstrated PFO-migraine association (5, 6)—it also carries inherent risks. Potential complications of PFO closure include device-related thrombosis, atrial arrhythmias, and residual shunting. Additionally, post-intervention antiplatelet or anticoagulant therapy is often required to mitigate thrombotic risk, which may introduce additional concerns such as bleeding tendencies or drug interactions. Nevertheless, after careful evaluation of the risks and benefits associated with the procedure and long-term pharmacotherapy, PFO closure may help improve quality of life by reducing dependence on migraine medications.

Three international randomised controlled clinical trials (MIST, PRIMA, and PREMIUM) have reported on the clinical efficacy of PFO closure in patients with migraine and PFO (7–9). The MIST trial found no significant differences in the primary endpoint (migraine cessation) or secondary endpoints (symptom improvement or quality of life enhancement). However, *post-hoc* analysis (excluding two outliers) revealed that the implant group exhibited greater reduction in monthly migraine days (a 37% decrease, from 6.0 to 3.8 days) compared to the sham group (26% decrease, from 5.0 to 3.7 days; *P* = 0.027). The PRIMA trial's primary endpoint (mean reduction in migraine days) and secondary endpoints (such as reductions in migraine attacks, quality of life measures, and depression scores) were negative. Nonetheless, *post hoc* analyses revealed significantly greater reductions in migraine with aura days (−2.4 vs. −0.6 days; *P* = 0.014) and attacks (−2.0 vs. −0.5; *P* = 0.0003) in the PFO closure group compared to the control group. The PREMIUM trial did not meet its primary endpoint (≥50% reduction in migraine reduction); however, PFO closure significantly reduced headache days (a secondary endpoint), with higher responder rates observed in the intervention group than in controls (49% vs. 23%, *p* = 0.015). Of note, 15.4% of patients with frequent aura achieved complete migraine cessation, compared to 2.5% of controls (*p* = 0.04).

Many studies have investigated the efficacy of PFO occlusion for headaches in adults; however, few have addressed whether PFO occlusion is effective in children with migraines. Although some studies have reported a positive correlation between PFO closure and migraine, there is currently no clear consensus regarding the selection of patients suitable for intervention nor how to define surgical indications. Moreover, no studies have yet addressed the relationship between hidden PFO closure and migraine improvement. As such, the present study analysed data from 35 Chinese children diagnosed with PFO combined with migraine (with or without aura), aiming to explore the efficacy of PFO occlusion to further analyse its possible mechanism(s) to provide a basis for identifying the most suitable candidates for surgical treatment.

## 2 Material and methods

### 2.1 Patient selection

Data from 35 children, including 13 males (37.1%) and 22 females (62.9%); aged 9–15 years (10.91 ± 2.07 years), diagnosed with PFO and migraines at the Tianjin Children's Hospital (Tianjin, China) between March 2020 and February 2024, were retrospectively collected and analysed.

Patients fulfilling the following criteria were included: migraine diagnosed by a neurologist according to the International Classification of Headache Disorders (ICHD-3). They were divided into two groups: migraine with aura (MA) and migraine without aura (MO). The inclusion criteria included the following: the diagnosis of PFO was confirmed by contrast transthoracic echocardiography (cTTE, using agitated saline solution) which demonstrates higher sensitivity than conventional transthoracic echocardiography (TTE) in detecting moderate-to-large right-to-left shunts; positive contrast-transcranial doppler ultrasonography (c-TCD), with grade Ⅲ shunt; previous regular anti-migraine drug treatment for >3 months had been ineffective; and occurrence of migraines that seriously affected the activities of daily living, leading to a strong desire for intervention.

Individuals diagnosed with headache with a clear cause, those with a migraine history <6 months, obvious abnormal coagulation function and other surgical contraindications, and contraindications to antiplatelet drugs were excluded.

### 2.2 c-TCD foaming test

**Figure 1:**
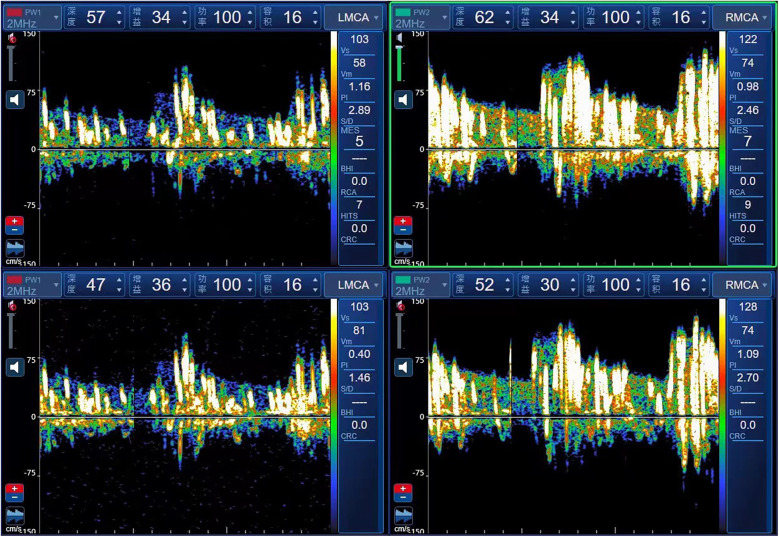
Positive contrast-transcranial Doppler ultrasonography test with grade III.

The c-TCD grading standard follows the 2,000 guidelines for detecting right-to-left shunt using an ultrasound contrast agent and transcranial Doppler sonography (10). The intervention procedure involved injecting a microvesicle contrast agent (a mixture of 9 ml saline and 1 ml air) through the elbow vein and insonating at least one middle cerebral artery (MCA) using TCD. If a right-to-left shunt is present, microbubbles can enter the left heart and systemic circulation through an abnormal pathway. Microbubbles signals entering the cerebral arteries can be monitored by TCD and are more easily detected during the Valsalva manoeuvre. If no microbubbles are detected at rest, the Valsalva manoeuvre (10 s duration, initiated 5 s post-injection) is repeated. According to the number of microbubbles, shunts are categorised into four grades: Grade 0, no microbubbles detected; Grade Ⅰ, 1–10 microbubbles; Grade Ⅱ, >10 microbubbles without a curtain effect; and Grade Ⅲ, embolic signal curtain or shower type. Children diagnosed with PFO and migraine were screened and referred to the Ultrasound Department for further examination. The grade III c-TCD foaming test is illustrated in Figure 1.

### 2.3 Saline contrast echocardiography

**Figure 2:**
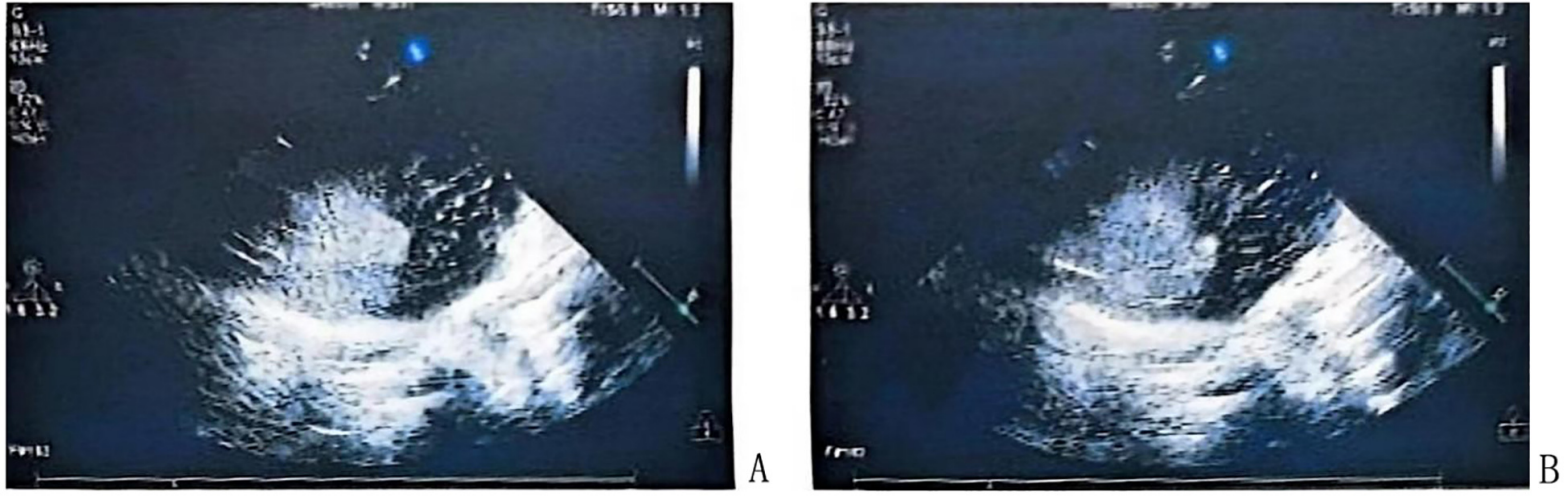
Saline contrast echocardiography in the apical four-chamber view **(A,B)** reveals abundant contrast microbubbles in the left atrium and left ventricle, respectively, confirming a large right-to-left shunt.

Children who tested positive in the c-TCD foaming test were referred to the Ultrasound Department for saline contrast echocardiography. A 10 ml syringe with 1 ml of air and another 10 ml syringe with 8 ml of saline were connected to a three-way stopcock, and 1 ml of venous blood was collected and mixed. The resulting mixture was immediately injected into the elbow veins. The Valsalva manoeuvre was performed, and the number of bubbles filling the left atrium was graded by observing four sections of the heart cavity: Grade 0 (no shunt), no bubbles observed in the left atrium; Grade I (small shunt), 1–10 bubbles; Grade Ⅱ (moderate shunt), 10–30 bubbles; and Grade Ⅲ (large shunt), >30 bubbles (11). Representative positive saline contrast echocardiography images showing a large shunt are presented in Figures 2A,B.

### 2.4 PFO occlusion

**Figure 3:**
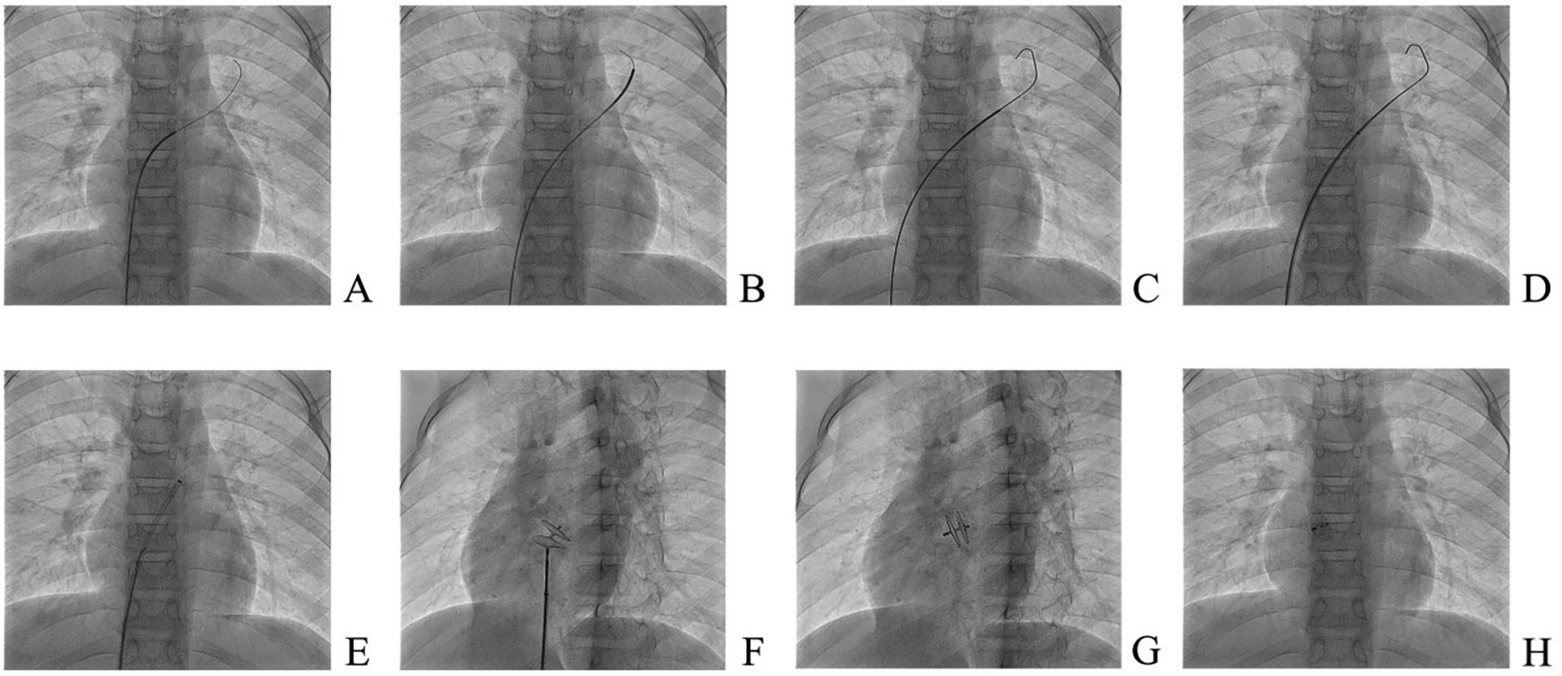
Patent foramen ovale occlusion. **(A)** The guidewire enters the left upper pulmonary vein under digital subtraction angiography (DSA) guidance; **(B)** a multifunctional catheter is advanced into the left upper pulmonary vein under DSA guidance; **(C)** the stiff guidewire is introduced into the left upper pulmonary vein under DSA; **(D)** the delivery sheath is sent to the left atrium along the stiff guidewire; **(E)** the delivery sheath is transferred to the left chamber; **(F)** the occluder is deployed in the oval hole; **(G)** the occluder is released in the left front oblique position and the occluder is in the tangential position; **(H)** The position of the occluder is satisfactory.

**Figure 4:**
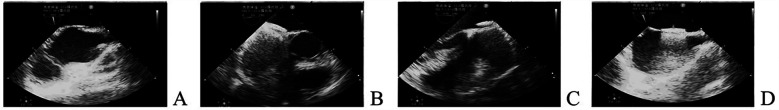
Ultrasound changes before and after intervention. **(A)** Ultrasound before plugging; **(B)** Short axial section of the great artery after closure; **(C)** Four-chamber view of the heart apex after closure; **(D)** Double-compartment section below the xiphoid process after closure.

Patients underwent intervention under general anaesthesia and transesophageal ultrasound monitoring using an 18/18 mm PFO occluder (Huayi Shengjie Co., Ltd., Beijing, China). After general anaesthesia, patients were positioned supine, disinfected, and covered with a towel. The right femoral vein (RFV) was punctured, a 6 Fr vascular sheath was inserted, and a 5 Fr multifunctional catheter was inserted through the RFV to the fossa ovalis. A loach guidewire was inserted through the foramen ovale into the left upper pulmonary vein, and a multifunctional catheter was inserted along the loach guidewire into the left upper pulmonary vein. The loach guidewire was then pulled out, a 260 cm 0.035-in exchange guidewire was sent through a 5 Fr multifunctional catheter and fixed in the left upper pulmonary vein, and the 6 Fr delivery sheath was transported along the guide wire to the left upper pulmonary vein opening. The inner sheath and guidewire were then pulled out and the occluder was sent along the long sheath to the left atrium. The left atrial umbrella was released, the right atrial umbrella was released into the right atrium, and the atrial interval was fastened. Intraoperative oesophageal ultrasound was used to observe whether the occluder had any effect on the surrounding structures. If there was no effect, the occluder was released and local pressure was applied to stop bleeding. An 18/18 mm occluder was used in all operations. Dual antiplatelet therapy with aspirin (3–5 mg/kg/d, max 100 mg) and clopidogrel (1 mg/kg/d, max 75 mg) was administered post-intervention for three months, followed by aspirin monotherapy for an additional three months. All antiplatelet medications were discontinued after six months. The PFO occlusion procedure in illustrated in Figure 3. Ultrasound changes before and after intervention are reported in Figure 4.

### 2.5 Data analysis

Basic information, clinical characteristics, pre-intervention examinations and evaluations, detailed intervention information, post-intervention occluder location, the degree of intra-atrial right-to-left shunt, length of hospital stay, post-intervention follow-up, and other related information were summarised. Standardised follow-up evaluations were conducted at 1 and 12 months after PFO closure, during which clinicians systematically assessed patients' headache diaries (including migraine attack frequency, duration, pain intensity, associated symptoms, and medication use), headache rating scales, and medical records through face-to-face consultations or telephone interviews. The following clinical outcomes were assessed as follows:

2.5.1 The NRS-11 requires children to directly express the intensity of pain with a specific number, Zero represents no pain, ten represents the most intense pain, and the intermediate values indicate varying degrees of pain.
2.5.2 The HIT-6 was used to evaluate the effects of pain, social role function, cognitive function, psychological abnormality, and vitality on the quality of life of children with headaches. The total score ranges from 36 to 78 points, with higher scores indicating a greater impact on the child's daily life.
2.5.3 The PedMIDAS was used to assess the total number of days in the past 3 months during which the child was unable to complete the following activities: work or school, housework, and family and social activities. The higher the score, the more severe was the impact.
2.5.4 The frequency of migraine(s) reflects the number of days per month the child experienced headaches.
2.5.5 The duration of each migraine attack refers to the average length of headache (in hours) in a day over the past three months.

During follow-up, staff neurologists evaluated all clinical outcome measures. Improvement in migraine after PFO occlusion was defined by the following: improvement in the assessment results of the migraine-related questionnaires; complete remission of migraine; and reduction of the frequency of migraine(s) by >50%.

### 2.6 Statistical analysis

SPSS version 23.0 (IBM Corporation, Armonk, NY, USA) was used for statistical analysis. Measurement data are expressed as mean ± standard deviation and compared using paired *t*-tests or rank-sum tests. Differences between groups were compared using *t*-tests or rank-sum tests. Count data are presented as frequencies and/or percentages (%) and compared using chi-square tests. A *p*-value <0.05 was considered statistically significant.

## 3 Results

The present study retrospectively analysed data from 35 children including 13 males (37.1%) and 22 females (71%), diagnosed with PFO and migraine who fulfilled the inclusion criteria. The age at onset ranged from 9 to 15 years (10.91 ± 2.07), and the disease duration ranged from 0.5–4 years (mean 1.39 ± 0.80). There were 12 cases of migraine with aura and 23 cases of migraine without aura. The main symptom was headache. The aura in migraine with aura included visual aura (9 cases), brainstem aura (3 cases), and motor aura (1 case, hemiplegic migraine). Primary outcome measures included NRS-11, HIT-6, PedMIDAS, migraine frequency, and duration of each migraine attack, with the following respective mean values: 5.91 ± 1.11, 60.34 ± 5.27, 86.06 ± 54.50, 17.06 ± 6.22 and 6.49 ± 2.59. Baseline characteristics of the children included in this study are summarised in Table 1.

**Table 1:** Clinical characteristics of children with PFO and migraine.

Different groups Baseline characteristics		MA (12 cases) *n* (%)	MO (23 cases) *n* (%)	Total (35 cases) *n* (%)
Age		11.67 ± 2.39	10.52 ± 1.77	10.91 ± 2.07
Gender	Male	4 (33.3)	9 (39.1)	13 (37.1)
	Female	8 (66.7)	14 (60.9)	22 (62.9)
Disease duration		1.18 ± 0.96	1.51 ± 0.68	1.39 ± 0.80
Clinical symptoms (aura)				
Visual aura		9 (75.0)	/	9 (25.7)
Brainstem aura		3 (25.0)	/	3 (8.6)
Motor aura		1 (8.3)	/	1 (2.9)
Comorbidities				
Ischemic stroke		1 (8.3)	0 (0.0)	1 (2.9)
Depression		1 (8.3)	0 (0.0)	1 (2.9)
Migraine Prophylactic Drugs				
Flunarizine		10 (83.0)	23 (100.0)	33 (94.3)
Topiramate		2 (16.7)	0 (0.0)	2 (5.7)
Auxiliary examinations				
Pre-intervention echocardiography	PFO (3–5 mm)	9 (75.0)	19 (82.6)	28 (80.0)
	Intact atrial septum	3 (25.0)	4 (17.4)	7 (20.0)
C-TCD foaming test	Positive, grade III	12 (100.0)	23 (100.0)	35 (100.0)
Saline contrast echocardiography	Positive, moderate shunt	3 (25.0)	8 (34.8)	11 (31.4)
	Positive, large shunt	9 (75.0)	15 (65.2)	24 (68.6)
Migraine-related scores				
NRS-11		6.67 ± 1.25	5.52 ± 0.77	5.91 ± 1.11
HIT-6		62.58 ± 6.86	59.17 ± 3.71	60.34 ± 5.27
PedMIDAS		94.17 ± 68.16	81.57 ± 43.76	86.06 ± 54.50
Migraine frequency (per month)		22.17 ± 6.83	14.39 ± 3.72	17.06 ± 6.22
Duration of each migraine attack (hours)		6.25 ± 3.49	6.61 ± 1.95	6.49 ± 2.59

Values are, *n* (%) or mean ± SD.

MA, migraine with aura; MO, migraine without aura; PFO, patent foramen ovale; c-TCD, contrast-enhanced transcranial doppler ultrasound; NRS-11, numerical rating scale-11; HIT-6: headache impact test-6; PedMIDAS, pediatric migraine disability assessment.

### 3.1 Results of transesophageal ultrasonography-guided PFO closure

All patients underwent PFO occlusion at the Cardiovascular Centre of Tianjin Children's Hospital. The entire operation was performed under digital subtraction angiography (DSA) guidance and transesophageal ultrasonography, and the occluder was implanted with a success rate of 100%. The occluder was in a good position, and the atrial horizontal shunt disappeared without a residual shunt. The mean length of hospitalisation was 3–4 days. There was one case of transient grade II type 1 atrioventricular block and one case of transient sinus bradycardia, all of which returned to normal after review. No serious peri-intervention complications or adverse events, including cardiac deterioration, cardiothoracic effusion, instrument thrombosis, stroke, or haemorrhage, occurred. During follow-up, all patients underwent TTE at 1-month post-intervention. At the 12-month follow-up, subsequent evaluation utilized c-TTE, as existing evidence indicates that complete endothelialization of the occluder typically requires 6 months. The results showed no instances of right-to-left shunt in any patient.

### 3.2 Improvement in migraine symptoms after PFO occlusion

Most patients exhibited varying degrees of improvement in migraine symptoms after PFO closure. At the 1-month follow-up after the intervention, 20 patients (57.1%) achieved complete remission, and 29 (82.9%) experienced a reduction in migraine frequency of >50%. At the 12-month follow-up, 28 patients (80%) achieved complete remission, and 32 (91.4%) experienced a reduction in migraine frequency of >50%. There were no cases of worsening migraine after PFO occlusion. The NRS-11, HIT-6, PedMIDAS, migraine frequency, and migraine duration of all the children three months before the intervention, at 1 month after the intervention, and at 12 months after the intervention are shown in Table 2, which demonstrates a significant decrease in the main clinical outcome indicators of migraines from baseline to 12 months after the intervention. Notably, there were statistically significant differences between pre-intervention and one-month post-intervention data, as well as between pre-intervention and 12-month post-intervention data (*p* < 0.05). One year after PFO closure, only one patient continued to use the prophylactic migraine medication flunarizine, whereas two patients used analgesics solely during migraine attacks.

**Table 2:** Comparison of migraine-related assessment scales before and after PFO closure.

Different time points Scale	Base lines	M1		M12	
		Scale	*p-*value	Scale	*p-*value
NRS-11	5.91 ± 1.11	**1.14** ± 1.48*	0.00	**0.51** ± 1.11*	0.00
HIT-6	60.34 ± 5.27	**40.63** ± 5.93*	0.00	**37.43** ± 3.99*	0.00
PedMIDAS	86.06 ± 54.50	**8.68** ± 13.60*	0.00	**4.17** ± 12.23*	0.00
Migraine frequency (per month)	17.06 ± 6.22	**3.26** ± 4.49*	0.00	**1.17** ± 2.63*	0.00
Duration of each migraine attack (hours)	6.49 ± 2.59	**1.21** ± 1.40*	0.00	**0.43** ± 0.99*	0.00

Values are mean ± SD. Within-group comparisons were performed using paired *t*-tests or Wilcoxon signed-rank tests.

M1, 1 month after intervention; M12, 12 months after intervention; NRS-11, numerical rating scale-11; HIT-6, headache impact test-6; PedMIDAS, pediatric migraine disability assessment.

The asterisk (*) on the bold values indicates statistically significant (*p* < 0.05) compared to baseline.

### 3.3 Improvement of migraine in aura and non-aura groups

**Figure 5:**
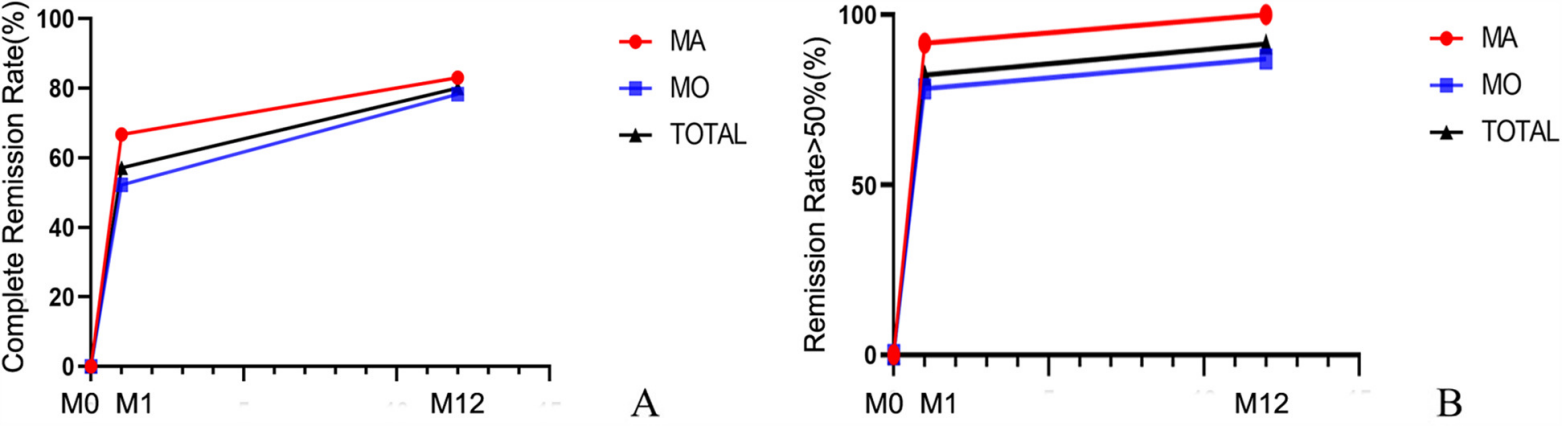
Changes in the remission rate of migraine with aura and migraine without aura over time. **(A)** Complete Remission Rate; **(B)** Remission Rate ≥50%. MA, Migraine with aura; MO, Migraine without aura; TOTAL, the overall patient group. M0, 3 months before the intervention; M1, 1 month after intervention; M12, 12 months after intervention.

Among the 35 children, 12 (34.3%) had migraine with aura and 23 (65.7%) had migraine without aura. There were no differences between the two groups in terms of sex and age. During the 12-month follow-up, 10 children (83%) in the aura group and 18 children (78.3%) in the non-aura group achieved complete remission of migraine. Additionally, 12 children (100%) in the aura group and 20 children (87%) in the non-aura group experienced a reduction in migraine frequency of >50%. There were no statistically significant differences between the aura and non-aura groups (Figure 5, Table 3).

**Table 3:** Remission rates of migraine with aura and migraine without aura at different time points after intervention.

Proportion (*n*/%) Groups (cases)	M1				M12			
	Complete cure		Relief rate >50%		Complete cure		Relief rate >50%	
	(*n*/%)	*p-*value	(*n*/%)	*p-*value	(*n*/%)	*p-*value	(*n*/%)	*p-*value
MA (12 cases)	8 (66.7)	0.49	11 (91.7)	0.64	10 (83)	1.0	12 (100)	0.54
MO (23 cases)	12 (52.2)		18 (78.3)		18 (78.3)		20 (87)	
TOTAL (35 cases)	20 (57.1)		29 (82.9)		28 (80)		32 (91.4)	

Values are *n* (%). Intergroup comparisons were performed using the Chi-square test or Fisher's exact test.

M1, 1 month after intervention; M12, 12 months after intervention; MA, migraine with aura; MO, migraine without aura. .

**Figure 6:**
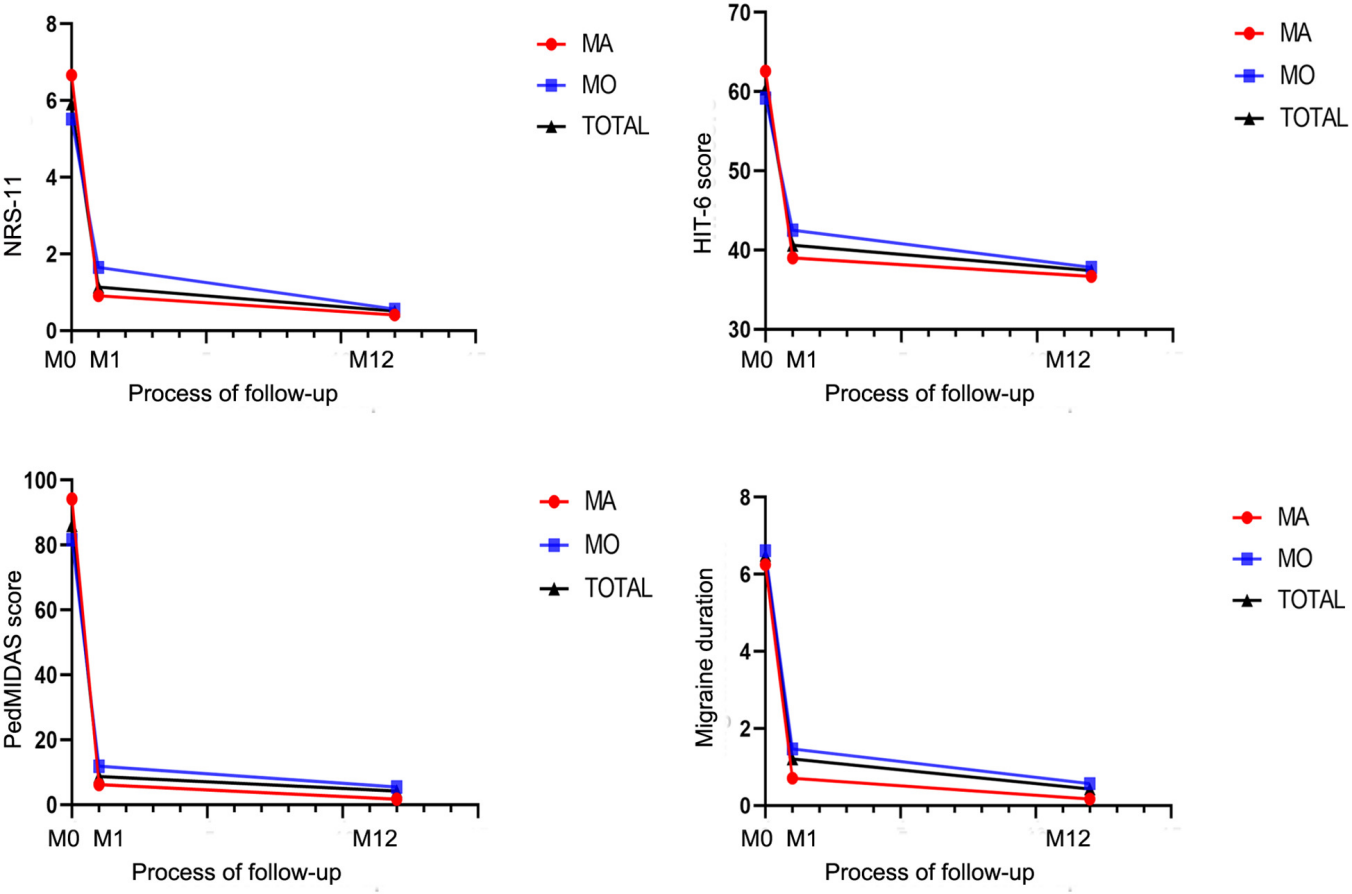
Changes in headache-related scores in children with migraine with aura and in those with migraine without aura after patent foramen ovale closure.

The reduction in NRS-11 at 12 months after intervention was more pronounced in the aura group than in the non-aura group (*P* < 0.05), while there were no statistically significant differences between the two groups in terms of HIT-6, PedMIDAS, and migraine duration (Figure 6, Table 4).

**Table 4:** Headache-related scores at different time points after patent foramen ovale closure in children with migraine with aura and in those with migraine without aura.

Groups Scales	MA			MO			Improvement differences between groups
	M0	M12	*p-*value	M0	M12	*p-*value	
NRS-11	6.67 ± 1.25	**0.42** ± 0.95*	0.002	5.52 ± 0.77	**0.57** ± 1.17**	0.000	0.026
HIT-6	62.58 ± 6.86	**36.67** ± 1.49*	0.002	59.17 ± 3.71	**37.83** ± 4.75**	0.000	0.067
PedMIDAS	94.17 ± 68.16	**1.67** ± 3.82*	0.002	81.64 ± 44.74	**5.47** ± 14.66**	0.000	0.572
Duration of each migraine attack (hours)	6.25 ± 3.49	**0.16** ± 0.37*	0.002	6.61 ± 1.95	**0.57** ± 1.17**	0.000	0.970

Values are mean ± SD. Within-group comparisons were performed using paired *t*-tests or Wilcoxon signed-rank tests, and intergroup comparisons were performed using independent samples *t*-tests or Mann–Whitney *U* tests.

MA, migraine with aura; MO, migraine without aura; M0, 3 months before the intervention; M12, 12 months after intervention; NRS-11, numerical rating scale-11; HIT-6, headache impact test-6; PedMIDAS, pediatric migraine disability assessment.

The asterisks (*/**) on the bold values indicate statistically significant differences compared to M0, with *p*-values of *p* < 0.05 and *p* < 0.001, respectively.

## 4 Discussion

Our study demonstrated significant therapeutic benefits of PFO closure in pediatric migraine patients with moderate-to-large right-to-left shunts. At the 1-month follow-up after intervention, 20 patients (57.1%) achieved complete remission, and 29 (82.9%) experienced a reduction in migraine frequency of >50%. At the 12-month follow-up, 28 patients (80%) achieved complete remission, and 32 (91.4%) experienced a reduction in migraine frequency of >50%. During the 12-month follow-up period, the headache-related scores decreased significantly compared to pre-intervention levels, with statistical significance. Both the aura and non-aura groups demonstrated high response rates (83% vs. 78.3% complete remission; 100% vs. 87% achieving >50% frequency reduction), with no statistically significant intergroup differences (Figure 5, Table 3). The reduction in NRS-11 at 12 months after intervention was more pronounced in the aura group than in the non-aura group (*P* < 0.05), while there were no statistically significant differences between the two groups in terms of HIT-6, PedMIDAS, and migraine duration. Moreover, no serious adverse events occurred, supporting the safety of the procedure. These findings support the potential therapeutic value of PFO closure in selected pediatric migraine patients, particularly in those demonstrating medication-refractory symptoms coupled with objectively confirmed moderate-to-large right-to-left shunts.

Migraine is a common disabling neurological disease with an incidence of approximately 10%–20% among school-age children, and gradually increases with age (12). Migraine symptoms vary with age. Preschool children often exhibit irritability, crying, and vomiting, whereas older children often experience bilateral or unilateral temporal pain (13). The foramen ovale is the channel through which blood flows from the right to the left atrium during the foetal period. With the development of the heart *in utero*, the primary and secondary septa of the foramen ovale gradually develop and overlap. The foramen ovale is necessary for transportation throughout the foetus's life, allowing oxygenated blood from the placenta to bypass the lungs and enter the systemic circulation of the embryo. In most infants, due to reduced pulmonary vascular resistance and higher pressure in the left atrium than in the right atrium after a few breaths, the foramen ovale functionally closes immediately after birth, generally reaching anatomical closure five to seven months after birth. If the foramen ovale is not closed after three years of age, it is termed “PFO”. The incidence of PFO in adults is approximately 25% (14). Although PFO is found in nearly half of the patients with migraine with aura, its frequency in patients with migraine without aura is similar to that in non-migraineurs. This suggests that PFO is particularly associated with migraines that include aura (15, 16).

Current evidence supports PFO closure in carefully selected younger patients with cryptogenic stroke when (1) no alternative stroke aetiology is identified, and (2) the PFO is associated with a large right-to-left shunt (17, 18). Although PFO appears to be beneficial, controversy persists regarding its use in the treatment of migraine (19). Three international randomised controlled clinical trials (MIST, PRIMA, and PREMIUM) failed to meet their primary endpoints, with only the PREMIUM trial achieving its secondary endpoint of significantly reducing headache days and demonstrating higher efficacy than the control group. However, *post-hoc* analyses revealed that PFO closure has potential benefits in reducing migraine frequency and improving quality of life, particularly in patients with migraine with aura (7–9). In contrast, Hildick-Smith et al. speculated that PFO occlusion was less effective than conservative treatment, and thus could only achieve a narrowly acceptable outcome (20). Therefore, the application of PFO closure in the treatment of migraine remains controversial, and its practical clinical application lacks strong research support. There are more data for adults and fewer for children.

The children included in this study were between 9 and 15 years of age. These children experienced long and sometimes severe headaches, with no clear cause, ineffective medical treatment, which was a strong and negative influence on their studies and life. Both the c-TCD foaming test and saline contrast echocardiography were positive, with the former showing Grade III and the latter demonstrating a moderate-to-large shunt. The decision to perform PFO closure in this study was made cautiously based on individualised clinical assessments, given the current lack of standardised guidelines for PFO closure indications in the paediatric population. These children had significant migraine relief after PFO occlusion.

Although three hypotheses have been proposed, the pathophysiological link between PFO and migraines remains unclear. First, the Paradoxical Embolism Hypothesis suggests that the right-to-left shunt (RLS) may allow microemboli (e.g., thrombi or platelet aggregates) to bypass pulmonary filtration, enter the cerebral circulation, and trigger cortical spreading depression (CSD), the electrophysiological correlate of the migraine aura (21). Key evidence includes thrombus formation within the PFO: Yan et al. identified the presence of *in situ* thrombi in patients with PFO who presented with cryptogenic stroke or migraine. More patients with thrombotic migraine experienced aura than those without (55.1% vs. 19.0%; *p* = 0.004) (22). These thrombi may originate from local endothelial dysfunction or stasis within the PFO channel rather than from deep vein thrombosis (DVT), as patients often lack conventional thrombotic risk factors (23, 24). Moreover, PFO closure demonstrates superior efficacy in migraine patients with intra-PFO thrombi, suggesting that embolism is a mechanistic contributor (22). By eliminating the shunt, PFO closure prevents the paradoxical embolisation of thrombotic material. Second, the Vasoactive Substance Hypothesis proposes that the RLS may allow serotonin and other vasoactive substances (such as neurotransmitters or endothelin) to bypass the lungs and not be metabolised by monoamine oxidase in the lungs but directly access the brain circulation. The arrival of large quantities of 5-hydroxytryptamine (5-HT) activates pain fibres around meningeal vessels, with the resultant release of pain mediators—which accompany the pain effect of serotonin—leading to vasodilation and further activation and sensitisation of the trigeminal sensory neurones. PFO closure restores pulmonary filtration of vasoactive substances, significantly reducing their direct stimulation of the central nervous system and, consequently, decreasing migraine frequency (21).Third, the Hypoxia Hypothesis suggests that the RLS caused by PFO may permit partially deoxygenated venous blood to bypass pulmonary oxygenation and enter systemic circulation. This may lead to transient cerebral hypoxia and impaired cerebral oxygen delivery, particularly during Valsalva manoeuvres. Hypoxia triggers cortical spreading depression (CSD) and induces migraine attacks (25, 26).

A meta-analysis has shown that 81% of migraine patients with aura experienced significant improvement in headache after PFO closure, while only 63% of those without aura showed improvement, indicating that patients with migraine with aura benefit significantly more from PFO closure than those without aura (*P* = 0.03) (27). Compared with previous literature, the remission rate in this study was higher. The better efficacy may be attributed to the cautious selection of patients with migraine and PFO. In children with a moderate to large shunt volume, PFO closure can effectively prevent the direct entry of a significant number of thrombi or vasoactive substances into the cerebral circulation due to right-to-left shunting when intrathoracic pressure increases, thereby avoiding migraine attacks. Additionally, the improvement in blood oxygenation can reduce the occurrence of migraines. In children with a small shunt volume, the above mechanism is not necessarily the cause of migraines; therefore, the intervention outcome may not be satisfactory. Therefore, it is necessary to precisely determine the indications for intervention in patients with migraine combined with PFO. For children with severe headaches and positive c-TCD foaming test result showing grades III and saline contrast echocardiography test showing a moderate-large shunt, the outcomes may be better. The lack of statistical difference in migraine remission rates between the aura and non-aura groups may be related to the young age of the children, atypical symptoms, or the inability to effectively describe aura.

It should be noted that PFO closure was also performed in children with no significant PFO detected by echocardiography, but positive in the c-TCD foaming test and saline contrast echocardiography, indicating an abnormal moderate–to-large shunt of the foramen ovale. This is because in 20%–34% of the population, the primum and secundum septa do not fuse, leading to the formation of a valve. The PFO remains closed most of the time; however, behaviours such as sneezing, coughing, or straining may cause a brief and rapid increase in intrathoracic pressure and open the PFO. This residual and transient traffic between the right and left atria provides a conduit for blood clots, air, or vasoactive peptides to travel from the veins to the arterial circulation. Its sequelae include a range of clinical phenomena, including cryptogenic stroke, systemic thrombosis, migraine, and decompression sickness (19). Therefore, for children experiencing headache for >6 months, the cause of headache is excluded, the conservative treatment of neurological drugs is ineffective, and the study and life are greatly affected, even if there is no obvious shunt on echocardiography, it is recommended to further perform a c-TCD foaming test and saline contrast echocardiography to confirm the size of the shunt of the ovalis, so as to further evaluate whether there are surgical indications.

The present study had several limitations, including its single-centre, retrospective design, small number of patients, and lack of a control group. The placebo effect is prominent in migraine treatments, particularly in interventional trials. Placebo responses may operate through multiple mechanisms, including patient expectations, psychological suggestion, and increased confidence in the treatment. Although our study employed objective assessment tools (such as the NRS-11, HIT-6, and PedMIDAS), the scoring of these instruments necessarily relied, to some extent, on patients' subjective perceptions. However, the complete symptom relief rate in this study increased from 57.1% at one month post-intervention to 80% at 12 months, a pattern inconsistent with typical placebo effects, which tend to diminish over time. Additionally, the PREMIUM trial demonstrated that a >50% reduction in migraine days was achieved in only 15% of patients in the pharmacological treatment group, whereas the complete relief rate in our study (80%) far exceeded this benchmark. Although this study adopted a retrospective design, it provides valuable clinical evidence for evaluating the efficacy of PFO closure in the treatment of migraines in children. The relatively small sample size may limit the statistical power of the analysis, particularly regarding comparisons between migraine with aura and without aura. This limitation reflects the current lack of standardised guidelines and consensus on the indications for PFO closure in paediatric populations, leading to cautious patient selection. Future studies with long-term follow-up are recommended to assess the long-term efficacy and safety of PFO closure. Alternatively, multicentre, prospective, large-scale, randomised controlled trials should be conducted to validate the therapeutic effects of PFO closure in children with migraines.

In conclusion, our study demonstrated that PFO closure can be an effective treatment option for paediatric patients with refractory migraines and moderate-to-large shunts, as evidenced by c-TCD and saline contrast echocardiography. Although the exact pathophysiological mechanisms linking PFO and migraines remain unclear, our findings suggest that careful patient selection, particularly in those with moderate-to-large shunts and migraine with aura, may lead to improved outcomes. Further research is needed to establish standardised guidelines for PFO closure in paediatric populations and to elucidate the underlying mechanisms.

## Data Availability

The raw data supporting the conclusions of this article will be made available by the authors, without undue reservation.
